# Trends in mortality in patients with systemic autoimmune rheumatic diseases (SARD) during the COVID-19 pandemic in Mexico

**DOI:** 10.1007/s00296-023-05371-w

**Published:** 2023-06-22

**Authors:** Pamela Munguía-Realpozo, Claudia Mendoza-Pinto, Ivet Etchegaray-Morales, Juan Carlos Solis-Poblano, Jorge Ayón-Aguilar, Edith Ramírez-Lara, Jacsiry Orbe-Sosa, Socorro Méndez-Martínez, Mario García-Carrasco

**Affiliations:** 1 Systemic Autoimmune Diseases Research Unit, Specialties Hospital UMAE-CIBIOR, Instituto Mexicano del Seguro Social, Puebla, Mexico; 2 Department of Rheumatology, Medicine School, Benemérita Universidad Autónoma de Puebla, Calle 13 Sur 2702, Los Volcanes, 72420 Puebla, Mexico; 3 Department of Haematology, Specialties Hospital UMAE, Instituto Mexicano del Seguro Social, Puebla, Mexico; 4 Coordinación Médica de Investigación en Salud, Instituto Mexicano del Seguro Social, Delegación Puebla, Puebla, Mexico; 5 Coordinación de Planeación y Enlace Institucional, Instituto Mexicano del Seguro Social, Delegación Puebla, Puebla, Mexico

**Keywords:** Systemic autoimmune rheumatic diseases, Age-standardized mortality rate, COVID-19 pandemic, Annual percentage change

## Abstract

The study aimed to analyze the influence of the COVID-19 pandemic on mortality rates in patients with systemic autoimmune rheumatic diseases (SARD) in Mexico. We selected SARD-related deaths using National Open Data and Information from the Ministry of Health, Mexico, and ICD-10 codes. We assessed the observed compared to the predicted mortality values for 2020 and 2021, employing trends from 2010 to 2019 with joinpoint and prediction modelling analyses. Among 12,742 deaths due to SARD between 2010 and 2021, the age-standardized mortality rate (ASMR) increased significantly between 2010 and 2019 (pre-pandemic) (annual percentage change [APC] 1.1%; 95% CI 0.2–2.1), followed by a non-significant decrease during the pandemic period (APC 13.9%; 95% CI 13.9–5.3). In addition, the observed ASMR of 1.19 for 2020 for SARD and of 1.14 for 2021 were lower than the predicted values of 1.25 (95% CI 1.22–1.28) for 2020 and 1.25 (95% CI 1.20–1.30) for 2021. Similar findings were identified for specific SARD, mainly systemic lupus erythematosus (SLE), or by sex or age group. Interestingly, the observed mortality rates for SLE in the Southern region of 1.00 in 2020 and 1.01 in 2021 were both significantly greater than the predicted values of 0.71 (95% CI 0.65–0.77) in 2020 and 0.71 (95% CI 0.63–0.79). In Mexico, the observed SARD mortality rates were not higher than the expected values during the pandemic, except for SLE in the Southern region. No differences by sex or age group were identified.

**Supplementary Information:**

The online version contains supplementary material available at 10.1007/s00296-023-05371-w.

## Introduction

Systemic autoimmune rheumatic diseases (SARD) comprise a heterogeneous group of disorders characterized by an altered immune response leading to inflammation and systemic involvement [1]. Despite recent advances in therapies, the burden of SARD remains high despite recent advances in therapies [2]. In Mexico, some SARD have experienced a decrease in mortality rates [3], others have exhibited an increase in mortality and hospitalizations [4–6].

Three years ago, the World Health Organization (WHO) announced that the world was experiencing a pandemic as a consequence of severe acute respiratory syndrome coronavirus (SARS-CoV-2) infection, denominating the COVID-19 pandemic. To date, this pandemic has had impressive and expansive consequences on the population.

The COVID-19 pandemic affected access to health services, with frequent avoidance of office visits and laboratory testing, particularly in the early months of the pandemic [7, 8]. Interruptions in taking disease-modifying antirheumatic drugs also commonly occur without the advice of a physician [8]. Racial and ethnic differences in COVID-19 outcomes have been reported in patients with SARD. African-American, Latin American, and Asian individuals have increased odds of hospitalization and ventilatory support [9].

Nationwide, indicative data leading to the precise allocation of resources and preventive actions for the most affected population are lacking. Thus, the objective of our study was to evaluate the trends and influence of COVID-19 on the mortality rates of subjects with SARD in Mexico from the years 2010–2021, with a specific assessment of SARD. In addition, variations in mortality rates according to age, sex, and geographic region among SARD patients were evaluated.

## Methods

### Study design and data sources

In this epidemiological study, data were acquired from the website of the National Institute of Statistics and Geography [INEGI] through the General Directorate of Health Information (DGIS) Open Access of the Ministry of Health (SSA) in Mexico [10]. This database collects death records from death certificates from across the country. The data were updated in December 2021. In addition, data from Mexican National Population Council (CONAPO) projections were gathered [11]. Age, sex, and geographic region were obtained. Geographic regions were categorized according to the INEGI groups. This study was approved by our Institutional Review Board (R-2022-2106-011).

### Population selection

We selected information on SARD-related deaths among Mexican subjects aged 15 years or above from January 1, 2010, to December 31, 2021. Data on all-cause of mortality were collected using the 10th edition of the International Classification of Diseases (ICD-10). We included the following SDAR: systemic lupus erythematosus (SLE) (M32*), systemic sclerosis (SSc) (M34*), idiopathic inflammatory myopathies (IIM) (M33*), rheumatoid arthritis (RA) (M05-M06*), and antineutrophil cytoplasmic antibody (ANCA)-associated vasculitis (AAV) (M31*). Death certificates that did not provide geographical or sociodemographic data were excluded.

### Statistical analysis

Descriptive analyses are presented as frequencies with percentages. Age-standardized mortality rates (ASMR, per 100,000 inhabitants) were calculated using the age framework (15–75+ years) from 2010 to 2021 in the Mexican population using the direct standardization method. Initially, we determined the national trends of mortality in subjects with SARD by conducting a joinpoint analysis (piecewise linear regression) [12]. For the overall trend, one or more pieces of the annual percentage change (APC) with 95% confidence intervals (95% CI) were analyzed using a Monte Carlo permutation test. Then, in order to estimate the predicted mortality values in 2020 and 2021, derived from mortality values from 2010 to 2019 to contrast with the observed values, we carried out a predictive analysis employing linear models between years and ASMR. The predicted rate was compared with the observed rate and evaluated to determine whether the observed rate overlapped with the 95% CI of the predicted value. The SPSS Statistics (version 25; SPSS, Chicago, IL, USA) time series model with the expert modeler choice was utilized to predict the future trend of SARD mortality. Additionally, we performed a subgroup analysis for sex (female and male) and geographic region (Northern, Midwest, Central, and Southern). Subgroups with ≤ 20 deaths may have led to inaccurate calculations for mortality rates; therefore, these were not performed. All calculations were conducted using the joinpoint analysis (version 4.9.0.0) issued by the United States National Cancer Institute (http://surviellance.cancer.gov/joinpoint). A 2-sided *p* < 0.05 was considered statistically significant.

## Results

### Study population

Overall, 13,143 deaths among patients aged ≥ 15 years with SARD were recorded between 2010 and 2021. SLE was the most frequent common cause of death (67.8%) followed by SSc (19.5%), PM/DM (6.4%), RA (3.3%), and AAV (3.0%) (Table S1). The majority of deaths were in females (83.9%) overall and in every SARD. The majority of deaths occurred in persons aged between 15 and 54 years (67.4%) at the time of death for overall and specific SARD, with the exception of RA (28.5%). There were notable variations in the distribution of mortality among the geographic regions. Death due to SARD occurred overwhelmingly among the residents of the central region.

## Influence of COVID-19 pandemic on ASMR

### Overall analysis for SARD ASMR

**Fig. 1:**
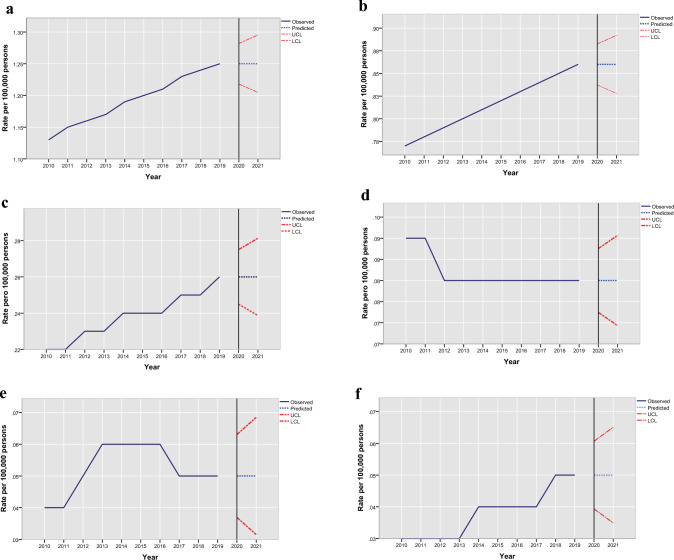
**a** All-cause age-standardized mortality for systemic autoimmune rheumatic diseases in Mexico in 2010–2021 with comparisons between observed (blue line) vs. predicted (blue dots) mortality for 2020 and 2021 based on 2010–2019 trend for overall. **b** All-cause age-standardized mortality for systemic autoimmune rheumatic diseases in Mexico in 2010–2021 with comparisons between observed (blue line) vs. predicted (blue dots) mortality for 2020 and 2021 based on 2010–2019 trend for SLE. **c** All-cause age-standardized mortality for systemic autoimmune rheumatic diseases in Mexico in 2010–2021 with comparisons between observed (blue line) vs. predicted (blue dots) mortality for 2020 and 2021 based on 2010–2019 trend for SSc. **d** All-cause age-standardized mortality for systemic autoimmune rheumatic diseases in Mexico in 2010–2021 with comparisons between observed (blue line) vs. predicted (blue dots) mortality for 2020 and 2021 based on 2010–2019 trend for IIM. **e** All-cause age-standardized mortality for systemic autoimmune rheumatic diseases in Mexico in 2010–2021 with comparisons between observed (blue line) vs. predicted (blue dots) mortality for 2020 and 2021 based on 2010–2019 trend for RA. **f** All-cause Age-standardized mortality for systemic autoimmune rheumatic diseases in Mexico in 2010–2021 with comparisons between observed (blue line) vs. predicted (blue dots) mortality for 2020 and 2021 based on 2010–2019 trend for AAV. *AAV* antineutrophil cytoplasmic antibody (ANCA)-associated vasculitis, *IIM* idiopathic inflammatory myopathies, *LCL* lower confidence limits, *RA* rheumatoid arthritis, *SLE* systemic lupus erythematosus, *SSc* systemic sclerosis, *UCL* upper confidence limits

The ASMR (per 100,000 inhabitants) for SARD remained stable from 1.16 in 2010 to 1.16 in 2021, generating an average APC of 0.0% for 2010–2021 (95% CI − 1.8 to 1.8; *p* = 0.99). The ASMR and APC for mortality in Mexico in patients with SARD are shown in Table 1. Interestingly, in the trend-segment evaluation, we identified a significant uptrend of 1.3 (95% CI 0.3–2.3) from 2010 to 2019 and then a stable trend from 2019 to 2021. The observed ASMR of 1.23 for 2020 for SARD and of 1.16 for 2021 were nearly similar to the predicted values of 1.30 (95% CI 1.26–1.34) for 2020 and 1.30 (95% CI 1.25–1.30) for 2021 (Fig. 1a). Similar segmental trends have been observed in patients SLE patiens. Although the ASMR increased slightly from 0.77 in 2010 to 0.80 in 2021, the average APC was 0.3% (95% CI − 1.1 to 1.8; *p* = 0.654). The observed ASMR of 0.83 in 2020 and of 0.83 in 2021 were slightly lower than the predicted values of 0.86 (95% CI 0.84–0.88) in 2020 and of 0.86 (95% CI 0.83–0.89) in 2021 (Fig. 1b). The ASMR trend for SSc remained steady from 2010 to 2021 at an average APC of 0.3 (95% CI − 5.1 to 6.1) and without significant variation (Fig. 1c). The observed ASMR for 2020 and 2021 for SSc were marginally lower than the predicted rates for SSc (0.24 vs. 0.26 for 2020 and 0.24 vs. 0.26 for 2021). Similarly, mortality-rate trends were found for IIM, RA and AAV (Figs. 1d, e and f), in which the ASMR trend was stable from 2010–2021 at an average APC of − 3.7 (95% CI − 7.5 to 0.4), 0.3 (95% CI − 7.7 to 8.9), − 0.7 (95% CI − 11.6, 11.5) respectively. The observed ASMR for 2020 and 2021 for IIM, RA and AAV were slightly lower than the predicted values (0.07 vs. 0.8 for 2020 and 0.06 vs. 08 for 2021 for IIM, and 0.04 vs. 0.05 for 2020; 0.04 vs. 05 for 2021 for RA and 0.04 vs. 0.05 for 2020 and 0.02 vs. 0.5 for AAV).

**Table 1:** All-cause age standardized mortality rate (ASMR) and annual percentage change (APC) in mortality in Mexico subjects with overall SARDs and by SARDs type

	Deaths (age-standardized rate per 100,000)			Average APC (95% CI)	Trend segment		p value
	2010 (Pre-pandemic reference epoch)	2020 (Pandemic epoch 1)	2021 (Pandemic epoch 2)	2010–2021	Year	APC (95% CI)	
Overall	938 (1.16)	1164 (1.23)	1114 (1.16)	0.0 (− 1.6 to 1.7)	2010–2019	**1.3 (0.3–2.3)**	**0.018**
					2019–2021	− 5.6 (− 15.4 to 5.3)	0.253
SLE	650 (0.77)	817 (0.83)	788 (0.80)	0.3 (− 1.1 to 1.8)	2010–2019	**1.2 (0.4–2.1)**	**0.009**
					2019–2021	− 3.6 (− 11.8 to 5.3)	0.361
RA	29 (0.04)	44 (0.04)	42 (0.04)	0.3 (− 7.7 to 8.9)	2010–2014	12.5 (− 10.9 to 42.2)	0.271
					2014–2021	− 6.2 (− 13.6 to 1.9)	0.112
IIM	69 (0.09)	78 (0.07)	51 (0.06)	− 3.7 (− 7.5 to 0.4)	2010–2019	− 0.9 (− 3.2 to 1.4)	0.363
					2019–2021	− 15.0 (− 34.0 to 9.5)	0.174
SSc	164 (0.22)	225 (0.24)	233 (0.24)	0.3 (− 5.1 to 6.1)	2010–2019	1.7 (− 1.4–5.0)	0.233
					2019–2021	− 5.9 (− 33.2 to 32.7)	0.689
AAV	26 (0.03)	38 (0.05)	25 (0.02)	− 0.7 (− 11.6 to 11.5)	2010–2019	**7.7 (1.2–14.6)**	**0.026**
					2019–2021	− 31.0 (− 66.3 to 41.29)	0.260

Temporal trend analysis was performed using joinpoint analysis. APC and p values were calculated using the Monte Carlo permutation test

Values p < 0.05 are given in bold

*AA* antineutrophil cytoplasmic antibody (ANCA)-associated vasculitis, *IIM* idiopathic inflammatory myopathies, *RA* rheumatoid arthritis, *SARDs* systemic autoimmune rheumatic diseases, *SLE* systemic lupus erythematosus, *SSc* systemic sclerosis

### Subgroup analysis

#### By sex

As shown in Table 2, all-cause mortality for SARD remained stable across both sexes during the study period; however, segment trend variations were identified. The APC significantly increased in females from 2010 to 2019 (1.0; 95% CI 0.1–1.9), followed by a non-significant downward trend from 2019 to 2021. Similar mortality trends were observed in patients with SLE, while APC significantly increased in males from 2010 to 2019 (6.0; 95% CI 0.1–12.2), without change after that year. The observed ASMR rates were marginally lower than the expected levels for SARD in females (1.90 vs. 2.02 in 2020 and 1.79 vs. 2.02 in 2021). For other SARD, such as IIM, SSc, and RA, there were no segment-trend variations across both sexes during the study period and the observed ASMR values. Similarly, no differences were identified between the observed and predicted rates in IIM, SSc, RA, and AAV.

**Table 2:** All-cause ASMR and APC in mortality in Mexico subjects with SARDs, by SARDs type and by sex

	Deaths (age-standardized rate per 100,000)			Average APC (95% CI)	Trend segment		p value
	2010 (Pre-pandemic reference epoch)	2020 (Pandemic epoch 1)	2021 (Pandemic epoch 2)	2010–2021	Year	APC (95% CI)	
Overall							
Female	768 (1.84)	990 (1.90)	945 (1.79)	− 0.2 (− 1.8 to 1.3)	2010–2019	**1.0 (0.1–1.9)**	**0.031**
					2019–2021	− 5.7 (− 14.5 to 3.9)	0.194
Male	144 (0.36)	174 (0.36)	169 (0.35)	− 0.3 (− 3.8 to 3.3)	2010–2019	0.4 (− 1.6 to 2.4)	0.673
					2019–2021	− 3.1 (− 22.1 to 20.6)	0.744
SLE							
Female	570 (1.33)	705 (1.40)	678 (1.32)	− 0.1 (− 1.8 to 1.7)	2010–2019	**1.1 (0.2–2.1)**	**0.029**
					2019–2021	− 5.3 (− 14.9 to 5.5)	0.272
Male	80 (0.21)	112 (0.24)	110 (0.24)	1.1 (− 2.0 to 4.3)	2010–2012	− 0.5 (− 17.8 to 20.5)	0.951
					2012–2021	1.5 (− 0.3 to 3.3)	0.086
RA							
Female	21 (0.05)	37 (0.05)	29 (0.05)	0.6 (− 5.9 to 7.6)	2010–2014	3.0 (− 13.8 to 23.0)	0.711
					2014–2021	− 0.7 (− 7.9 to 7.1)	0.832
Male	8 (0.02)	7 (0.02)	13 (0.02)	− 2.3 (− 18.7 to 17.3)	NA	NA	NA
					NA	NA	NA
IIM							
Female	47 (0.07)	50 (0.05)	41 (0.04)	− 4.5 (− 9.1 to 0.4)	2010–2019	− 0.2 (− 2.9 to 2.6)	0.878
					2019–2021	− 21.5 (− 42.0 to 6.2)	0.100
Male	22 (0.22)	28 (0.17)	10 (0.17)	− 2.4 (− 14.9 to 12.0)	NA	NA	NA
SSc							
Female	130 (0.33)	198 (0.35)	197 (0.35)	0.3 (− 2.8 to 3.6)	2010–2016	2.7 (− 2.4 to 8.0)	0.259
					2016–2021	− 2.4 (− 8.1 to 3.6)	0.372
Male	34 (0.08)	27 (0.07)	36 (0.07)	− 1.6 (− 10.0 to 7.5)	2010–2019	− 0.1 (− 4.7 to 4.7)	0.955
					2019–2021	− 8.1 (− 46.9 to 59.3)	0.728
AAV							
Female	15 (0.03)	21 (0.3)	11 (0.2)	− 2.0 (− 19.8 to 19.7)	2010–2019	7.7 (− 3.7 to 20.5)	0.160
					2019–2021	− 36.1 (− 81.3 to 118.2)	0.417
Male	11 (0.03)	17 (0.04)	14 (0.03)	0.4 (− 9.4 to 11.2)	2010–2019	**6.0 (0.1–12.2)**	**0.048**
					2019–2021	− 21.4 (− 58.1 to 47.3)	0.394

Values p < 0.05 are given in bold

*AAV* antineutrophil cytoplasmic antibody (ANCA)-associated vasculitis, *APC* annual percentage change, *ASMR* age-standardized mortality rate, *IIM* idiopathic inflammatory myopathies, *RA* rheumatoid arthritis, *SARDs* systemic autoimmune rheumatic diseases, *SLE* systemic lupus erythematosus, *SSc* systemic sclerosis

#### By geographic region

Subgroup analysis for geographic regions in Mexico found a significant increase in ASMR from 2013 to 2021 among decedents in the southern region for SLE (APC, 5.0; 95% CI 1.0–9.1) (Table 3). The increased mortality rates observed for SLE in the southern region from 1.00 in 2020 to 1.01 in 2021 were both significantly greater than the predicted levels in 0.71 (95% CI 0.65–0.77) in 2020 and 0.71 (95% CI 0.63–0.79). No other significant variations were identified during the pandemic period for overall SARD or specific SARD such as SSc. Analyses for IIM, RA and AAV for ASMR mortality segments were not performed because of the small sample sizes for these conditions.

**Table 3:** All-cause age-standardized mortality rate (ASMR) and annual percentage change (APC) in mortality in Mexico subjects with SARDs, by SDAR type and by geographic region

	Deaths (age-standardized rate per 100,000)			Average APC (95% CI)	Trend segment		p value
	2010 (Pre-pandemic reference epoch)	2020 (Pandemic epoch 1)	2020 (Pandemic epoch 2)	2010–2021	Year	APC (95%)	
Overall							
Northern	237 (1.22)	282 (1.08)	224 (0.98)	− 1.9 (− 5.3 to 1.5)	2010–2019	− 0.2 (− 2.2 to 1.7)	0.780
					2019–2021	− 9.1 (− 26.5 to 12.4)	0.322
Midwest	212 (1.18)	259 (1.15)	258 (1.15)	− 0.2 (− 2.8 to 2.4)	2010–2012	− 2.0 (− 16.2 to 14.7)	0.733
					2012–2021	0.2 (− 1.3 to 1.6)	0.807
Central	358 (1.22)	458 (1.22)	461 (1.20)	− 0.1 (− 2.0 to 1.7)	2010–2016	1.4 (− 1.4 to 4.3)	0.276
					2016–2021	− 2.0 (− 5.5 to 1.7)	0.242
Southern	105 (0.76)	165 (1.00)	171 (1.01)	2.6 (− 0.6 to 6.0)	2010–2016	4.0 (− 0.9 to 9.1)	0.096
					2016–2021	1.0 (− 5.2 to 7.6)	0.718
SLE							
Northern	172 (0.84)	200 (0.82)	162 (0.75)	− 1.1 (− 5.8 to 3.8)	2010–2019	0.8 (− 1.9 to 3.6)	0.502
					2019–2021	− 9.3 (− 32.5 to 22.0)	0.463
Midwest	143 (0.78)	190 (0.88)	183 (0.83)	0.5 (− 2.6 to 3.7)	2010–2019	**1.9 (0.1–3.7)**	**0.042**
					2019–2021	− 5.3 (− 21.9 to 14.9)	0.528
Central	262 (0.87)	324 (0.94)	322 (0.88)	0.1 (− 2.1 to 2.5)	2010–2019	**1.6 (0.3–2.9)**	**0.025**
					2019–2021	− 6.0 (− 18.3 to 8.2)	0.335
Southern	73 (0.53)	103 (0.75)	121 (0.78)	3.6 (− 1.0 to 8.5)	2010–2013	0.2 (− 15.9 to 19.5)	0.976
					2013–2021	**5.0 (1.0–9.1)**	**0.020**
RA							
Northern	12 (0.05)	10 (0.03)	6 (0.03)	− 4.2 (− 11.6 to 3.8)	NA	NA	NA
Midwest	7 (0.05)	8 (0.04)	16 (0.06)	− 8.8 (− 17.5 to 0.9)	NA	NA	NA
Central	9 (0.03)	17 (0.03)	14 (0.02)	− 3.0 (− 11.9 to 6.8)	NA	NA	NA
Southern	1 (0.01)	9 (0.03)	6 (0.03)	1.0 (− 9.7 to 13.0)	NA	NA	NA
IIM							
Northern	15 (0.08)	23 (0.06)	11 (0.05)	− 5.5 (− 15.3 to 5.6)	NA	NA	NA
Midwest	21 (0.12)	12 (0.04)	10 (0.04)	2.2 (− 9.3 to 15.1)	NA	NA	NA
Central	20 (0.07)	28 (0.05)	17 (0.05)	− 2.2 (− 16.3 to 14.2)	NA	NA	NA
Southern	13 (0.08)	15 (0.09)	13 (0.09)	6.5 (− 19.3 to 40.6)	NA	NA	NA
SSc							
Northern	38 (0.21)	49 (0.16)	45 (0.15)	− 3.1 (− 8.0 to 2.0)	2010–2013	6.6 (− 12.5 to 30.0)	0.468
					2013–2021	− **6.6 (**− **10.3 to **− **2.6)**	**0.006**
Midwest	41 (0.22)	49 (0.17)	49 (0.18)	− 1.9 (− 7.6 to 4.1)	2010–2019	− 3.3 (− 6.6 to 0.1)	0.058
					2019–2021	4.6 (− 27.3 to 50.5)	0.779
Central	67 (0.25)	89 (0.22)	108 (0.23)	− 0.6 (− 10.8 to 10.7)	2010–2019	− 2.1 (− 8.2 to 4.3)	0.450
					2019–2021	6.6 (− 44.6 to 105.2)	0.824
Southern	18 (0.15)	38 (0.16)	31 (0.15)	− 0.4 (− 12.1 to 12.9)	NA	NA	NA
AAV							
Northern	7 (0.04)	6 (0.02)	4 (0.02)	− 4.9 (− 20.1 to 13.3)	NA	NA	NA
Midwest	8 (0.05)	12 (0.04)	7 (0.04)	− 1.7 (− 14.0 to 12.3)	NA	NA	NA
Central	9 (0.03)	17 (0.04)	13 (0.03)	1.6 (− 6.6 to 10.6)	NA	NA	NA
Southern	2 (0.01)	3 (0.01)	1 (0.01)	− 6.4 (− 29.4 to 24.1)	NA	NA	NA

Values p < 0.05 are given in bold

*APC* annual percentage change, *ASMR* age-standardized mortality rate, *IIM* idiopathic inflammatory myopathies, *RA* rheumatoid arthritis, *SARDs* systemic autoimmune rheumatic diseases, *SLE* systemic lupus erythematosus, *SSc* systemic sclerosis

No significant differences in mortality rates among age groups were identified during the study period for overall SARD and specific SARD (Table S2). However, the majority of deaths recorded were in the age group of 15–54 years for SARD, SLE, IIM and AAV.

## Discussion

In this population-based analysis, we evaluated the trends of mortality for SARD and for five specific SARD (SLE, IIM, SSc, RA and AAV) from 2010 to 2021 to establish any possible influence of the COVID-19 pandemic on the deaths of patients with these disorders.

Surprisingly, we did not find an increase in the observed all-cause mortality among patients with SARD during the 2019–2021 period, which was not larger than the values predicted from the pre-pandemic trend, except for SLE in the Southern region of the country. These findings were also found across sex and age groups. However, for most SARD cases, the number of deaths during the study period was higher in the younger population. Our findings were similar to those described recently in a nationwide population-based study in another Latin American country, Brazil [13], which showed no change in the trends of overall mortality from SLE. In contrast, in a nationwide analysis using observed and predicted evaluations from the United States, the authors identified an excess of psoriasis and psoriatic arthritis (inflammatory chronic diseases) mortality during the COVID-19 pandemic [14].

The SARS-CoV-2 infection affected 763 million people and has been responsible for severe affectation in 300 million, in 3.3 million new cases and nearly 6.8 million deaths globally [15]. In Mexico, according to the existing literature, there is excess mortality in the context of the COVID-19 pandemic, which is attributed mainly to the high burden of cardiometabolic disorders in Mexico [16]. In addition, heterogeneity in the burden of mortality during the COVID-19 pandemic has been identified in our country [17]. Our findings identified a diversified effect of the pandemic across geographic regions in Mexico and found that the southern region of Mexico was the sole region in which the observed mortality rates outpaced the predicted values in SLE mortality during the pandemic period. This finding confirms that this region experienced higher death rates from some SARD, as previously reported [4, 5]. This is partly due to higher sociodemographic inequalities and limited access to health care.

Humans possess defense mechanisms against viral infections, including interferon (IFN). Rheumatic diseases are associated with high levels of IFN and viral infection. Inhibition of IFN may be desirable in certain autoimmune diseases, specifically SLE [18]. Type-I IFN immunity is essential for adequate protection, and harmful mutations as well as neutralizing auto-antibodies against IFN have seldom been identified, particularly in the severe manifestations of COVID-19 [19].

Recently, we learned that among patients with SLE and RA, there could be an increase in the risk and severity of COVID when there are pre-existing neutralizing autoantibodies against IFN-I, which are associated with severe COVID in at least 10–20%; in general, the discontinuation of therapy appears to participate in disease flares [20].

Despite continuing deaths from COVID-19 in 2020 and 2021, the mortality from overall SARD and specific SARD remained stable, and the observed mortality rates were even lower than expected in Mexico. The reasons for these findings are unclear; however, some potential explanations are provided. First, reductions in the diagnosis of SARD with some possible displaced mortality and with some patients with SARD dying of COVID-19 before they had completed the final diagnosis of SARD, the latter complex conditions. Second, the deaths recorded as SARD, which were reduced during the pandemic period, may have been due to another cause, as the Mexican national vital statistics recorded only the underlying cause of death but not the contributing cause. Third, the majority of SARD deaths occurred in the younger population (aged 15–54 years). Considering that the older subjects were more prone to die from COVID-19 during the pandemic, this might reduce the number of deaths recorded as any SARD in the elderly, limiting the overall death in patients with SARD. Finally, the negative effects of pandemic control measures on mortality, such as delays in medical care, were probably more than offset by certain positive effects, such as personal hygiene and the implementation of social distancing, reducing some infections, an important risk factor for mortality in SARD, mainly in Latin American patients [21]. In this context, temporal variations in the occurrence of the leading cause of death originating from the COVID-19 pandemic in Mexico were analyzed, and some cause groups had notable declines in the expected pre-pandemic mortality, such as infectious, malignant skin, and musculoskeletal diseases (-5.0%) [22]. In addition, a multinational survey reported that patients with IIM had fewer COVID-19 cases than healthy controls, likely because of the protective behaviors adopted by this vulnerable population, including physical distancing and shielding [23]. Of 3502 patients with rheumatic diseases in patients inhabiting more than 19 Latin American countries, by means of a survey, more than one half of the patients had comorbidities, including hypertension. Antirheumatic therapy was discontinued in 23.4% of patients, and approximately 15% interrupted their comorbidity-associated treatment during the pandemic [24].

The strengths of this study are that, to the best of our knowledge, this is the first study to evaluate the impact of COVID-19 on SARD in Mexico. We standardized all mortality analyses by age, which provided a suitable comparison across Mexican regions with varied population structures. We used a nationwide registry with joinpoint analysis. However, this study has several limitations that should remind us to be considered when interpreting our findings. First, the inherent risk of bias in retrospective studies is applicable to our analysis. Second, we focused on trend mortality analysis, which is unable to quantify the exposure of risk factors that might contribute to the change in SARD tendencies. In addition, in our national database, only all-cause disease-specific data were available, making it impossible to analyze the specific impact of factors such as disease flares and treatments. Third, the case definition for each SARD-related death as the underlying cause of death was obtained from ICD-10 codes in contrast to better-quality clinical data, which is the reason for the level of diagnostic uncertainty and potential misclassification. However, ICD, which produces ICD-10 codes, was planned to foster worldwide equivalence in the acquisition and classification of information on mortality [25]. Notably, mortality data derived from ICD-10 are regularly utilized to determine mortality trends in epidemiological research evaluations of SARD [4, 5, 26, 27]. Fourth, miscoding of the cause of death cannot be ruled out using an administrative database, which may lead to underestimation. Finally, in our national dataset, only all-cause disease-specific causes of death were accessible, preventing further evaluation of the specific influence of determinants such as infections and other morbidities, including kidney disease and end-stage renal disease in patients with SLE. In agreement with our results, the current evidence does not strongly suggest that having immune-mediated inflammatory diseases increases the risk of developing severe COVID-19 [28]. However, although we did not observe any differential mortality in the COVID-19 period, our data raise concerns for undertreating SARD in the pandemic era, which may translate into worse outcomes in the long term. More research is needed to understand whether the patients not presenting to the healthcare system may have improved their compliance with medications in the COVID-19 era or whether they were slowly deteriorating at home. Additionally, the COVID-19 mass vaccination program in our country began in February 2021, which may have influenced our findings by 2021.

## Conclusions

Our epidemiological population-based study in Mexico provided data on all-cause mortality rates in SARD patients during the COVID-19 pandemic. This study demonstrated a significantly lower observed mortality rate in terms of expected values during the COVID-19 pandemic for the overall SARD. However, the observed mortality rate was significantly higher than that expected for patients with SLE in the Southern region of Mexico. Our study did not find other variations among sexes, age groups, or other geographic regions. Our results can aid in clinical practice and public health actions, considering the observed mortality trends in Mexico. We also motivate further epidemiological analysis, mainly in the long term and considering confounders, to evaluate these trends for other nationalities to provide a brief overview of the regional approach and policy-decision markers for the current pandemic.

### Supplementary Information

Below is the link to the electronic supplementary material.

Supplementary file1 (DOCX 18 KB)

## Data Availability

The National open data and information from the Ministry of Health, Mexico can be accessed through this website: http://www.dgis.salud.gob.mx/contenidos/basesdedatos/Datos_Abiertos_gobmx.html.
