# Correction to: Trends in mortality in patients with systemic autoimmune rheumatic diseases (SARD) during the COVID-19 pandemic in Mexico

**DOI:** 10.1007/s00296-024-05625-1

**Published:** 2024-06-10

**Authors:** Pamela Munguía-Realpozo, Claudia Mendoza-Pinto, Ivet Etchegaray-Morales, Juan Carlos Solis-Poblano, Jorge Ayón-Aguilar, Edith Ramírez-Lara, Jacsiry Orbe-Sosa, Socorro Méndez-Martínez, Mario García-Carrasco

**Affiliations:** 1 Systemic Autoimmune Diseases Research Unit, Specialties Hospital UMAE-CIBIOR, Instituto Mexicano del Seguro Social, Puebla, Mexico; 2 Department of Rheumatology, Medicine School, Benemérita Universidad Autónoma de Puebla, Calle 13 Sur 2702, Los Volcanes, Puebla, 72420 Mexico; 3 Department of Haematology, Specialties Hospital UMAE, Instituto Mexicano del Seguro Social, Puebla, Mexico; 4 Coordinación Médica de Investigación en Salud, Instituto Mexicano del Seguro Social, Delegación Puebla, Puebla, Mexico; 5 Coordinación de Planeación y Enlace Institucional, Instituto Mexicano del Seguro Social, Delegación Puebla, Puebla, Mexico

**Rheumatology International (2023) 43:1611–1619**

10.1007/s00296-023-05371-w

The article “Trends in mortality in patients with systemic autoimmune rheumatic diseases (SARD) during the COVID-19 pandemic in Mexico” written by Pamela MunguíaRealpozo, Claudia MendozaPinto, Ivet EtchegarayMorales, Juan Carlos SolisPoblano, Jorge AyónAguilar, Edith RamírezLara, Jacsiry OrbeSosa, Socorro MéndezMartínez, Mario GarcíaCarrasco was originally published electronically on the publisher’s internet portal on 22nd June 2024 without open access. With the author(s)’ decision to opt for Open Choice the copyright of the article changed on 10th June 2024 to © The Author(s) 2024 and the article is forthwith distributed under a Creative Commons Attribution 4.0 International License, which permits use, sharing, adaptation, [50] distribution and reproduction in any medium or format, as long as you give appropriate credit to the original author(s) and the source, provide a link to the Creative Commons licence, and indicate if changes were made. The images or other third party material in this article are included in the article’s Creative Commons licence, unless indicated otherwise in a credit line to the material. If material is not included in the article’s Creative Commons licence and your intended use is not permitted by statutory regulation or exceeds the permitted use, you will need to obtain permission directly from the copyright holder. To view a copy of this licence, visit http://creativecommons.org/licenses/by/4.0.

The original article has been corrected.

