# Supplementary material for: Trends in mortality in patients with systemic autoimmune rheumatic diseases (SARD) during the COVID-19 pandemic in Mexico
Source: Rheumatol Int. 2023 Jun 22;43(9):1611–9. doi: 10.1007/s00296-023-05371-w (PMC11162978; doi:10.1007/s00296-023-05371-w)
Supplement: Supplementary file 1 — Supplementary file1 (DOCX 18 KB) [file 296_2023_5371_MOESM1_ESM.docx]

**Supplementary material**

**Table S1.** Characteristics of systemic autoimmune rheumatic diseases related all cause of death in Mexico overall and by type, 2010-2021.

|  | **SARDs** | **SLE** | **RA** | **IIM** | **SSc** | **AAV** |
| --- | --- | --- | --- | --- | --- | --- |
| **Total** | 13143 | 8931 | 442 | 852 | 2517 | 401 |
| **Age at death** |  |  |  |  |  |  |
| 15-54 | 8858 (67.4%) | 6956 (77.9%) | 126 (28.5%) | 481 (56.5%) | 1056 (41.9%) | 239 (59.6) |
| ≥ 55 | 4285 (32.6%) | 1975 (22.2%) | 316 (71.5) | 371 (43.5%) | 1461 (58.1%) | 162 (40.4) |
| **Sex** |  |  |  |  |  |  |
| Female | 11027 (83.9%) | 7758 (86.9%) | 323 (73.1%) | 609 (71.5%) | 2127 (84.5%) | 210 (52.4%) |
| Male | 2116 (16.1%) | 1173 (13.1%) | 119 (26.9%) | 243 (28.5%) | 390 (15.5%) | 191 (47.6%) |
| **Geographic region** |  |  |  |  |  |  |
| Northern | 2992 (22.8%) | 2055 (23.1%) | 104 (23.5%) | 199 (23.3%) | 562 (22.3%) | 72 (18.0%) |
| Midwest | 2980 (22.7%) | 2066 (23.1%) | 105 (23.8%) | 165 (19.4%) | 539 (21.4%) | 105 (26.2%) |
| Central | 5463 (41.6%) | 3717 (41.6%) | 174 (39.4%) | 333 (39.1%) | 1047 (41.6%) | 192 (47.9%) |
| Southern | 1708 (12.9%) | 1093 (12.2%) | 59 (13.3%) | 155 (18.2%) | 369 (14.7%) | 32 (7.9%) |

AAV: antineutrophil cytoplasmic antibody (ANCA)-associated vasculitis; IIM: idiopathic inflammatory myopathies; RA: rheumatoid arthritis; SARDs: systemic autoimmune rheumatic diseases; SLE: systemic lupus erythematosus; SSc: systemic sclerosis.

**Table S2.** All-cause ASMR and APC in mortality in Mexico subjects with SARDs, by SARDs type and by age group region

|  | **Deaths (age-standardized rate per 100,000)** | | | **Average APC (95% CI)** | **Trend segment** | | **p value** |
| --- | --- | --- | --- | --- | --- | --- | --- |
|  | **2010 (Pre-pandemic reference epoch)** | **2020 (Pandemic epoch 1)** | **2020 (Pandemic epoch 2)** | **2010-2021** | **Year** | **APC (95% CI)** |  |
| **Overall** |  |  |  |  |  |  |  |
| 15-54 | 708 (1.22) | 772 (1.20) | 683 (1.11) | -0.8 (-2.5-0.9) | 2010-2019 | 0.7 (-0.3-1.7) | 0.146 |
|  |  |  |  |  | 2019-2021 | -7.4 (-16.8-3.1) | 0.134 |
| ≥ 55 | 230 (1.67) | 418 (2.24) | 456 (2.24) | 2.7 (-0.3-5.8) | 2010-2016 | **5.1 (0.6-9.9)** | **0.031** |
|  |  |  |  |  | 2016-2021 | -0.1 (-5.8-5.9) | 0.965 |
| **SLE** |  |  |  |  |  |  |  |
| 15-54 | 551 (0.93) | 620 (0.79) | 551 (0.79) | -1.4 (-4.0-1.2) | 2010-2013 | -4.5 (-13.6-5.5) | 0.309 |
|  |  |  |  |  | 2013-2021 | -0.2 (-2.4-2.0) | 0.803 |
| ≥ 55 | 620 (0.66) | 197 (1.02) | 237 (1.05) | 4.4 (-0.5-9.4) | 2010-2016 | 6.0 (-1.2-13.7) | 0.092 |
|  |  |  |  |  | 2016-2021 | 2.5 (-6.7-12.5) | 0.558 |
| **RA** |  |  |  |  |  |  |  |
| 15-54 | 8 (0.01) | 17 (0.02) | 15 (0.03) | 6.2 (-5.6-19.5) | NA | NA | NA |
| ≥ 55 | 21 0.16) | 27 (0.15) | 27 (0.13) | -1.8 (-11.4-8.8) | 2010-2014 | 12.4 (-14.5-47.9) | 0.344 |
|  |  |  |  |  | 2014-2021 | -9.1 (-19.0-2.1) | 0.936 |
| **IIM** |  |  |  |  |  |  |  |
| 15-54 | 40 (0.07) | 39 (0.6) | 25 (0.04) | -4.3 (-9.3-0.9) | 2010-2019 | -0.2 (-2.8-2.4) | 0.833 |
|  |  |  |  |  | 2019-2021 | -20.7 (-43.1-10.4) | 0.141 |
| ≥ 45 | 29 (0.20) | 39 (0.17) | 26 (0.16) | -2.1 (-13.4-10.7) | 2010-2019 | -1.0 (-7.6-6.1) | 0.749 |
|  |  |  |  |  | 2019-2021 | -7.0 (-56.2-97.5) | 0.826 |
| **SSc** |  |  |  |  |  |  |  |
| 15-54 | 89 (0.15) | 74 (0.12) | 79 (0.12) | -2.4 (-11.0-6.9) | 2010-2019 | -0.5 (-5.5-4.7) | 0.825 |
|  |  |  |  |  | 2019-2021 | -10.7 (-49.1-56.7) | 0.650 |
| ≥ 55 | 75 (0.53) | 151 (0.819 | 154 (0.82) | 4.0 (-2.0-10.4) | 2010-2012 | 15.3 (-19.8-65.8) | 0.385 |
|  |  |  |  |  | 2012-2021 | 1.7 (-1.6-5.1) | 0.276 |
| **AAV** |  |  |  |  |  |  |  |
| 15-54 | 20 (0.03) | 22 (0.03) | 13 (0.02) | -1.4 (-15.2-14.6) | NA | NA | NA |
| ≥ 55 | 6 (0.04) | 16 (0.08) | 12 (0.05) | 1.6 (-11.8-17.1) | NA | NA | NA |

AAV: antineutrophil cytoplasmic antibody (ANCA)-associated vasculitis; APC: annual percentage change; ASMR: age-standardized mortality rate; IIM: idiopathic inflammatory myopathies; RA: rheumatoid arthritis; SARDs: systemic autoimmune rheumatic diseases; SLE: systemic lupus erythematosus; SSc: systemic sclerosis.
